# Big data-drive agent-based modeling of online polarized opinions

**DOI:** 10.1007/s40747-021-00532-5

**Published:** 2021-09-17

**Authors:** Peng Lu, Zhuo Zhang, Mengdi Li

**Affiliations:** 1 Department of Sociology, Central South University, Changsha, China; 2 Center of Social System, Beijing Institute for General Artificial Intelligence, Beijing, China

**Keywords:** Online collective actions, Agent-based modeling, Expanded Ising model, Douban.com, Life cycles

## Abstract

Under the mobile internet and big data era, more and more people are discussing and interacting online with each other. The forming process and evolutionary dynamics of public opinions online have been heavily investigated. Using agent-based modeling, we expand the Ising model to explore how individuals behave and the evolutionary mechanism of the life cycles. The big data platform of Douban.com is selected as the data source, and the online case “NeiYuanWaiFang” is applied as the real target, for our modeling and simulations to match. We run 10,000 simulations to find possible optimal solutions, and we run 10,000 times again to check the robustness and adaptability. The optimal solution simulations can reflect the whole life cycle process. In terms of different levels and indicators, the fitting or matching degrees achieve the highest levels. At the micro-level, the distributions of individual behaviors under real case and simulations are similar to each other, and they all follow normal distributions; at the middle-level, both discrete and continuous distributions of supportive and oppositive online comments are matched between real case and simulations; at the macro-level, the life cycle process (outbreak, rising, peak, and vanish) and durations can be well matched. Therefore, our model has properly seized the core mechanism of individual behaviors, and precisely simulated the evolutionary dynamics of online cases in reality.

## Introduction

With the rapid development of Information and Communications Technologies (ICTs), more and more people are using internet to express opinions and exchange information [1]. And social media in the internet might facilitate open and critical debates that enhance the expression of the public [2]. Hence, the (mobile) internet becomes the main platform of individual expressions and opinion formations. Taking collective actions as the typical form, online public opinions are commonly stimulated or generated by specific events online. Public opinion is the collection or aggregation of individual feelings, cognitions, attitudes, emotions, and tendencies for online collective actions and information (or rumor) spreading [3]. With recent social media applications worldwide, such as social websites, chatting tools, online forums, and smart phone apps, the cyberspace becomes the major platform of public opinions and information diffusion [4]. Macroscopically, the mobile internet decentralizes political power. People online can make frank and serious criticisms of current social problems and political issues, without revealing their identities [5], which makes it more convenient for governments to understand emotions and viewpoints of the general public [6]. It also supports existing sociopolitical system both directly and indirectly, maintaining stability and legitimacy through online political expressions [7]. More and more researches find that online public opinion is bringing positive influences to the state-society relationship, especially in promoting political participation, forming public spheres, and reinforcing government accountability [8]; microscopically, it provides free expression channels for global netizens. Individuals are talking more about excited things online, such as food, traveling, sports, entertainment, and jobs [9]. Internet is used to browse breaking news, current events, and stories, and more public opinions can be formed. For non-political topics such as entertainment or daily events, more emojis are used. Under the social media time, the internet, therefore, becomes a major platform of online information diffusion and public opinion formation.

Online collective actions become one of the major research topics worldwide. For instance, numerous online collective behaviors have been frequently witnessed in China [10]. It indicates that massive online discussions lead to rapid formations of online public opinions, as well as online collective actions. China has the world’s largest netizen population, which is approximately 904 million in 2020 [11]. It continues to increase, and pervasive online discussions are rising, and online collective actions have greatly shaped the society. Chinese netizens use online collective actions to express their emotions, opinions or justice values. They are usually correlated to triggers, such as morals, politics, social inequality, or economic exploitation. Online discussions, collective petitions, voting, and even cyberspace attacks are common responsive forms of expressing their opinions and influencing others [12]. Neither state-owned nor market-controlled, online public opinions are characterized by free speech, universal access, and great inclusiveness [13]. Therefore, online collective actions give individuals a sense of collective justice, social identity and perceived efficacy [14]. However, free speech without boundaries and regulations can lead to ill-informed, ill-reasoned, or ill-behaved forms [15]. These extreme forms can easily cause social chaos and turbulences. Hence, how to model these irregular, complex, and stochastic public opinions [16], and how to predict them effectively [5], become the core aim of our work. Some proposed public opinion monitoring system (IPOMS) that adopts topic detecting and tracking methods [17], and others introduce cloud computing [18]. More researchers are using computers to do scientific experiments and simulations. They transform the real society into an evolutionary system with intelligent agents. This evolutionary system replaces “people” in reality with “artificial individuals”, which can reveal dynamic mechanisms between individual behaviors and macro-level social behaviors [19]. However, this paradigm always, first proposes possible theories, then collects data, and verifies them through calculations (based on past knowledge). After obtaining a deterministic mechanism, they establish new models for derivation. But this kind of experience and common sense may be incomplete, and it may lose important variables. The verifiability (data and models), data consistency, or simulation repeatability cannot be guaranteed as well [20]. Moreover, abandoning the exploration of causality and turning to correlations has been a new orientation of research paradigm [21]. Scientific research is entering the new age of big data or computational science. With the rapid development of information and network technology, massive activities have produced huge data, which forms the new infrastructure of “big data” [22]. We cannot only analyze trends of online public opinion through real-time monitoring and big data analysis. However, we should also design and explore the internal mechanisms of online cases. Nowadays, researchers no longer need models and assumptions, but they can use super-computing to directly analyze massive data to discover correlations and knowledge [23]. Big data can be much more accurate using the full sample, which realizes the analysis, calculation, and processing of online cases [24], and, therefore, enhances the practicality, objectivity, and accuracy. In this work, combining with big data analytics and agent-based modeling, which is easier, faster, and more comprehensive to analyze changes and connections of online cases than previous, and helps to accurately evaluate the integrity of online public opinions [25].

The current rapid developments of internet facilitate the public sphere formation [26], and related models of online public opinions have been proposed. Everyone is free to express online, and a new-generation of public sphere has emerged [27]. Online public opinions are formed, with significant impacts on the political voting [28], public policy [27], religions [29], and other social activities. This trend is especially obvious after the global rise of social media, such as Tik Tok, Twitter, Facebook, and Weibo. Related models focus on sentiment evolution, hotspot detection, trends monitoring, and evolution process. There are two categories: (A) detection algorithm. Based on mathematical algorithms, the big data dynamics of online public opinions can be mined and monitored. For instance, the hidden Markov model (HMM) combines online characteristics to predict online big data trends [30]. Besides, the K-means clustering and SVM methods [1], and cloud-computing can be applied as well to related monitoring and predictions [18]. BP neural network is used to analyze public opinions, using big data of networks. This method is optimized by genetic algorithms to predict the occurrence of crisis for online public opinions [31]. Recently, advanced machine learning algorithms such as deep learning, reinforcement learning, Chaos theory [16], and fuzzy neural networks [32] are also introduced into public opinions models; (B) agent-based models. Due to ethical or practical reasons, there are many experiments that cannot be carried out in the real world. Then, “synthetic environments” need to be created to simulate the complex system’s behavior of the real world [33]. ABM is a useful method to help us to understand the work of social mechanisms. And it provides a new method for our research to reduce the complexity of the social system or the natural system to an extent that allows us to guide our thinking, and at the same time, we can get an intuition of how certain changes in the system would affect its dynamics and outcome [34]. Agent-based models can simulate dynamic process, which improves existing formal models. For instance, the small-world model is used to model cyberspace emergence caused by netizens or agents [35]. The Galam model is used to predict the spreading of public opinions [36]. For new models, they also simulate the spreading of public opinions among netizens, news sites, and BBS agents [37]. Multi-agent models (netizens, leaders, government, and mass media) help to predict public opinion crisis [38]. The big data detecting and mining are static, but agent-based model is dynamic. Therefore, we combine cellular automata (CA) and Ising model, which can explore evolutionary dynamics of complex systems [39]. As discrete dynamic systems, CA model computes micro-level interactions to simulate macro-level patterns of complex system [40]. It can also do universal computations and stepwise simulations. More importantly, based on self-replication, it can predict future dynamics and trends [41]. Therefore, CA has been applied to multiple natural-social fields, with higher flexibility. As a typical CA model, the Ising model consists of numerous agents, who change status and interact with others, under given forces or rules [42]. Ising model explains how local interactions lead to collective actions, and, therefore, predicts evolutionary dynamics and phase transitions of the whole system. Ising model can reflect interactions of attitudes among netizens (agents), such as how they change their actions or attitudes under the influences of others. We all know that many models can accurately predict natural systems, but they are powerless to complicated social systems. Using the Ising model, we try to show how agents in the social system are interrelated, and how they are interacting with others, and this internal interaction eventually leads to the emergence of groups. Ising model can better represent the internal interaction of the social systems. For Ising model, the lattice is occupied by atoms of magnetic materials, and each atom has magnetic moments or directions (up and down). A small change in temperature or pressure may lead to large-scale changes of system, which is called phase transitions in physics [43]. Besides of simple expression and rich connotation, it can also simulate the criticality, which is widely witnessed in natural, social, and system sciences [44]. It can also explain racial segregation, business management, and language change [45]. For online human behaviors, Ising model is suitable to explore how neighbors shape individual perspectives online.

In Ising model, each small magnetic needle refers to each netizen, and the on–off states are likened to opposite views, opinions, or attitudes. Interactions between adjacent magnetic needles refer to local interactions. The temperature measures total environment, which refers to the percentage (probability) of netizens influenced by neighbors. Although scale-free networks have some advantages, they cannot replace the Ising model, which largely captures behavioral rules of netizen for many cases. For instance, online opinions for this target case are polarized, and people online often shift between two extreme cases (positive or negative). Compared with existing models, especially scale-free networks, the Ising model is, therefore, more responsive to changes in extreme views. It is more consistent to our target case, because: (a) scale-free network better captures key nodes and ordinary nodes (opinion leaders and followers). However, among Douban.com discussions, every online discussant is ordinary node, and no one can dominate the discussion and dissemination of information; (b) Ising model is more in line with the network opinion structure of BBS. For this case, the Douban.com discussions are similar to BBS. The opinion leaders have limited influence on others, because they can only see the viewpoints of the first few people [46]. The disappearance and change of individual viewpoint will not destroy the whole information network, which greatly avoids the vulnerability of scale-free network [47]; and (c) Ising model is good at modeling shifting and extreme viewpoints of individuals. For this case, viewpoints of many people online change relatively quickly and emotionally, and, in Douban.com, a tiny change of information may cause reversals of entire public opinions. Therefore, the results of the Ising model will have a better realistic fitness, than scale-free networks. Based on the Ising model, scholars simulate interactions between users’ self-identity (micro-level), user-user interactions (middle-level), and social environment influence (macroscopic-level), to model the rumor dynamics under the social network, and to investigate individual decisions within social networks [48]. Ising model can model dynamics of binary opinions, observe influences of structural characteristics of signed network, and explore structural balance of networks [49]. The influence of birth rates, sales of cell phones, the decay of applause to collective opinion shifts can be also analyzed with random field Ising model, which quantifies collective effects induced by imitations and social pressure [50]. Ising model can simulate and reflect real cases with multiple layers. For example, Liu et al. proposed Ising model for public opinions, with two kinds of neighbors on square lattice [51]. Under the periodic boundary, agents or netizens obey the majority rule. Finally, steady global patterns can be obtained. Then, they explore the influences of agents’ mobility, and the mobility will accelerate the formation of online public opinions [52]. With static and simple settings of agents, previous Ising models cannot explain the life cycle process of online cases [53]. Some previous models are far from reality [48], and some lack subjective settings of human beings [49]. To fix these pitfalls, we not merely expand the agent-based Ising model, but also combine empirical big data. The core aim of this paper is to achieve the highest matching or fitness between real cases and our agent-based simulations.

## Big data mining of real target case

We combine multiple methods, such as real case analysis, big data mining, agent-based modeling, and simulations. This work is to capture evolutionary dynamics and characteristics for online public opinions, such as durations, peaks, emotions, as well as the whole trends. According to change rate of netizens’ attitudes, we obtain and calculate basic trends of certain online cases. Targeting at the big data mining of real cases, we construct expanded Ising model and simulations to find the optimal solution, which best fits the big data features or trends of the real target cases.

### Big data mining

**Fig. 1:**
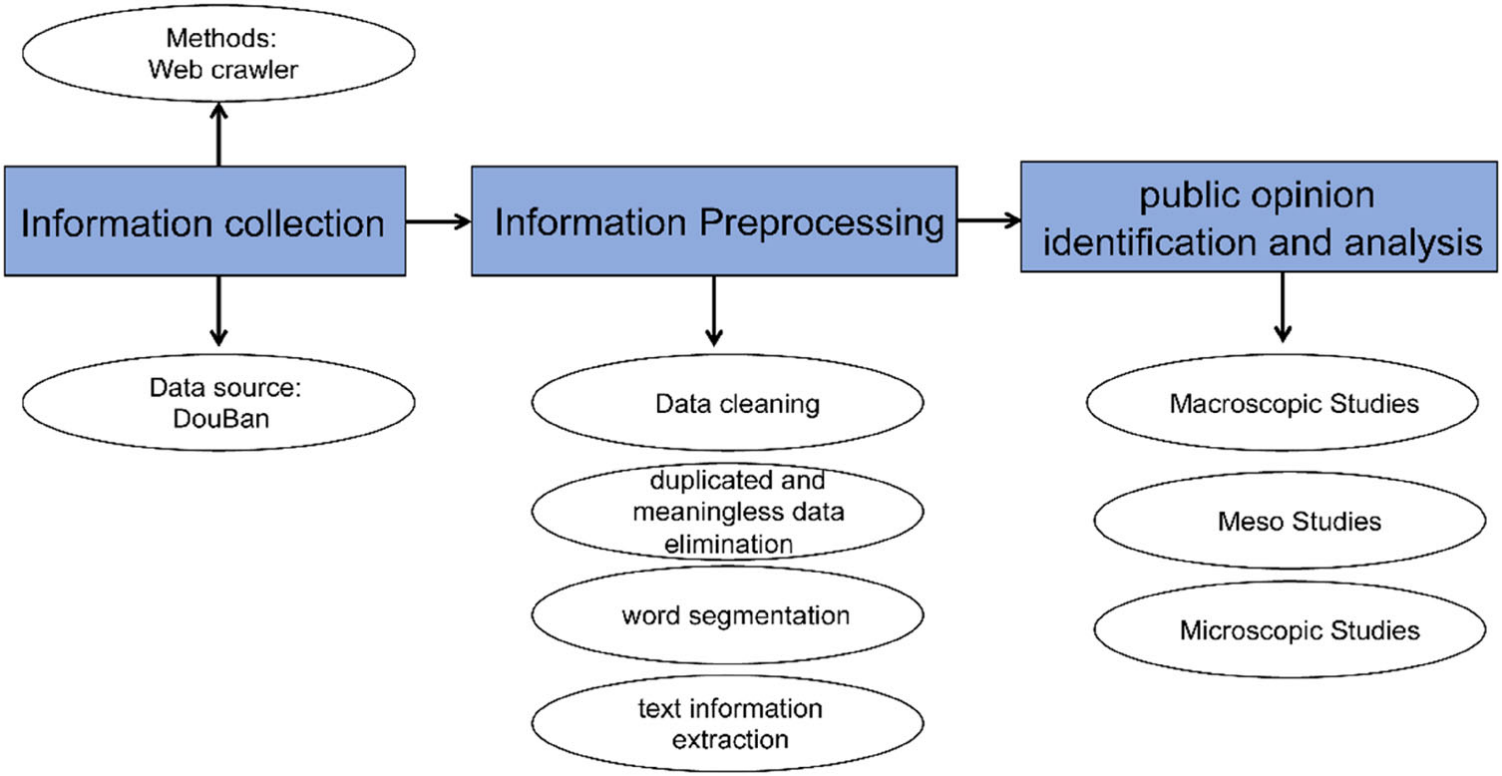
The framework of online public opinion mining. This system has three stages, such as information collection, information processing, public opinion identification, and big data analysis

Recently, it has been much easier for researchers to obtain big data worldwide. Facing massive internet contents, the big data are continuously generated daily, and reliable big data analytics becomes the critical skill [53]. Big data mining provides new challenges and opportunities for data managing and analyzing. Knowledge-discovery in databases (KDD), rough the same as big data mining, refers to the capability of extracting useful information from large datasets [54]. It uncovers complex patterns of hidden relationships, within large-scale data, and facilitates valuable predictions based on real-world observations. Its main purpose is unveiling huge, heterogeneous and complex big datasets, while maximizing our knowledge or insights into target domains [55]. Data mining method utilizes computational techniques to summarizes data into useful information, which can be used to predict future trends of online cases. This, in practice, helps governments, enterprises, or organizations to make knowledge-driven or data-driven decisions, and solve previously questions that is hard to figure out [56]. internet public opinion mining systems (IPOMS) includes the information collection layer, information processing layer, and identification and analysis layer [4]. Following three major layers, we collect big data of “NeiYuanWaiFang” case, as the target. We use big data mining methods to obtain data from the website (Douban.com) (Fig. 1).

### Case analysis and event detection

Online event detection is an important process in big data mining. It discovers new or latent online events within a continuous bunch of news [57]. Here, we use the “NeiYuanWaiFang” case. Since December 2019, the outbreak of pandemic was reported at Wuhan in China. On February 12nd, 2020, the WHO officially named this novel coronavirus as COVID-19. On January 25th, 2020, a famous Chinese writer, Ms. Fang Fang, released online version of her book, Fang Fang Diary, which records her daily observations in Wuhan under epidemic. Then, the diary was quickly renamed as Wuhan Diary, with a rapid publication rate. This dairy book contains controversial content. On April 12th, a Chinese singer (Bo Peep), released a song NeiYuanWaiFang to criticize her. This is obvious physical attack on Ms. Fang Fang, which leads to the outbreak of online “NeiYuanWaiFang” case. Many netizens were making discussions or comments on the writer as well as the song “NeiYuanWaiFang”. It seems that online commentaries were deeply polarized, which is suitable for Ising models. On the one hand, some netizens (score = 2 points, one star) supported free speech and free creation of Fang Fang Diary or Wuhan Diary, and they believed that healthy societies should have different voices; On the other hand, the netizens (score = 10 points, five stars) believe that this diary omits positive struggles against the virus in China and partially records painful losses. Based on personal influencing power, her diary with biased information spread worldwide, which harms China and the Chinese people. Automatic information monitoring on social networks facilitates investigations of public opinions [58]. In this work, we collect big data trends to analyze the big data features.

### Data acquisition of online public opinions

**Fig. 2:**
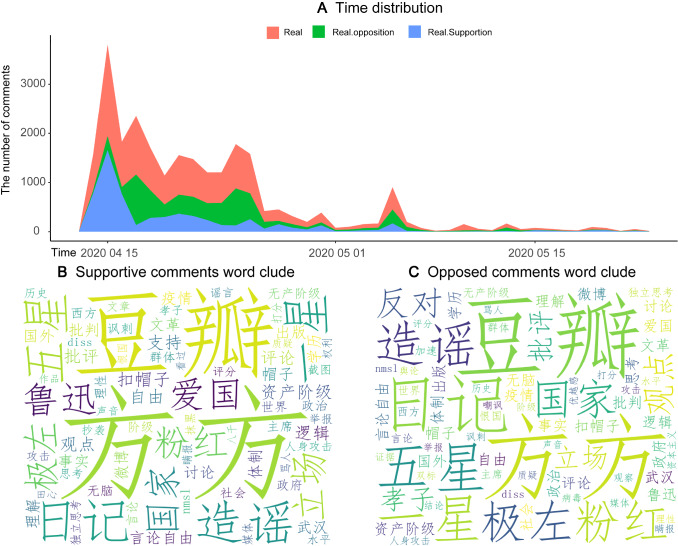
Time-series and content analysis of “NeiYuanWaiFang” case. In subfigure **A**, the red area represents daily volume of comments, the green represents daily oppositive comments, and the blue shows the trends of daily oppositive comments. Subfigures **B** and **C** are word clouds of both supportive and oppositive comments

As a huge database source, the web pages contain massive data, and we use multi-thread method to crawl more online webpages and store webpage sources [59]. The famous social networking website in China, the Douban.com has become the key platform where netizens (mostly readers, students and intellectuals) create, share, and comment global culture works (books, movies, documentaries, etc.), since 2005. With diverse data types and rich data volumes, the Douban.com is taken as the key barometer by western medias to obtain public opinions and attitudes of the Chinese people [60]. So, we choose Douban.com as the main data source. Generally, it includes three modules, such as short comments, long comments, and specific forums. Big data mining follows three steps: (A) data obtaining. First, we crawled the big data of real target case and collect information from Douban.com webpages, using the Python package “Beautiful Soup”. Since first released via Douban.com on April 12th, the song has sparked massive discussions, which forms the online “NeiYuanWaiFang” case. As it goes on, the discussion continues, with fewer and fewer people paying attention and expressing opinions. Hence, we obtain the whole life cycle, with a total volume of more than 13,500 discussions involved; (B) data preprocessing. We eliminate duplicated or meaningless discussion data. From April 13th to May 22nd, we record the life cycle of 40 days, with 13,036 valid discussions. The sentiment analysis of nature language processing (NLP) is supported by the Baidu platform with the sentiment knowledge enhanced pre-training (SKEP) algorithm, which makes substantial improvements on most datasets [61]. By calling the API of Baidu platform [62], we performed text extractions and word segmentations, to explore individual perspectives; (C) big data visualization. Figure 2A shows the time-serial trends of discussions or comments. The red line visualizes the overall trend. We also visualize both supportive and oppositive comments, which can be obtained through emotion analysis. We calculate sentiment values by uploading the captured text and using the sentiment analysis. This platform applies words segmentations and automatic matching of sentiment values, in terms of the distribution of word sentiment values. Each word has a sentiment value ${v}_{i}$, multiplied by the word frequency ${p}_{i}$, and added up as entire sentiment (${\sum }_{i=1}^{n}{p}_{i}\times {v}_{i}$) to obtain overall sentiment tendency [63]. In Fig. 2, the green line reflects oppositive trend, and the blue reflects supportive trend, during the whole life cycle. Figure 2B and C provides word-clouds of supportive and oppositive comments. The Python package “Jieba”, a common segmentation tool for Chinese characters, is used to divide supportive and oppositive comments into single Chinese words. In the segmentation processing, the stop words were filtered on the basis of general word database of Harbin Institute of Technology. Stop words include functional, meaningless high-frequency words, such as “functional words” and “lexical words” to reduce the index volume and improve the effect of retrieval, thus constructing an effective high-dimensional sparse text matrix. Then, we apply the segmented words to construct the relevant word cloud. Finally, we carry out online public opinions and analysis to lay the foundation for constructing our model; and (D) statistical of sentiments. Table 1 provides statistical descriptions of high-frequency words, for both supportive and oppositional words in comments. In total, we all have 6244 supportive discussions (comments) and 6792 oppositive discussions (comments). Hence, supportive opinions and oppositive opinions are well balanced, in terms of the size. We also calculated the discussion rate for each topic words by dividing the main vocabulary of a certain comment by the total number of posts. In supportive discussions, “Truth” is a word that appears more frequently (2.88%), and “logic” appears 179 times, accounting for about 2.87% of the total number of supportive discussions. Among oppositive discussions, “pink” is the most frequent vocabulary in the comments (3.68%), and the word “diss”, as a distinct oppositive discussion in Chinese, also appeared 64 times (0.94%).

**Table 1:** Data on commentary terms

Supportive			Oppositive		
Topic words	Count	Percent (*n* = 6244) (%)	Topic words	Count	Percent (*n* = 6792) (%)
Truth	180	2.88	Pink	250	3.68
Logic	179	2.87	Dutiful son	188	2.77
Discussion	136	2.18	Leftists	158	2.37
Rationally	85	1.36	NMSL (curse)	138	2.03
Rights	80	1.28	Hate the country	136	2.03
Independent thinking	71	1.14	Anencephaly	70	1.03
Expression freedom	72	1.15	Diss	64	0.94

## Agent-based expanded Ising model

The overall trend and both supportive and oppositive comments of individuals are key indicators of online public opinions, for the target case of our Ising model and simulations. The agent-based model is applied to investigate individual behaviors and macro-level patterns. Proven to be suitable and feasible, we expand Ising Model in cellular space, neighborhoods, cellular states, and the changing rules.

### Cellular space

**Fig. 3:**
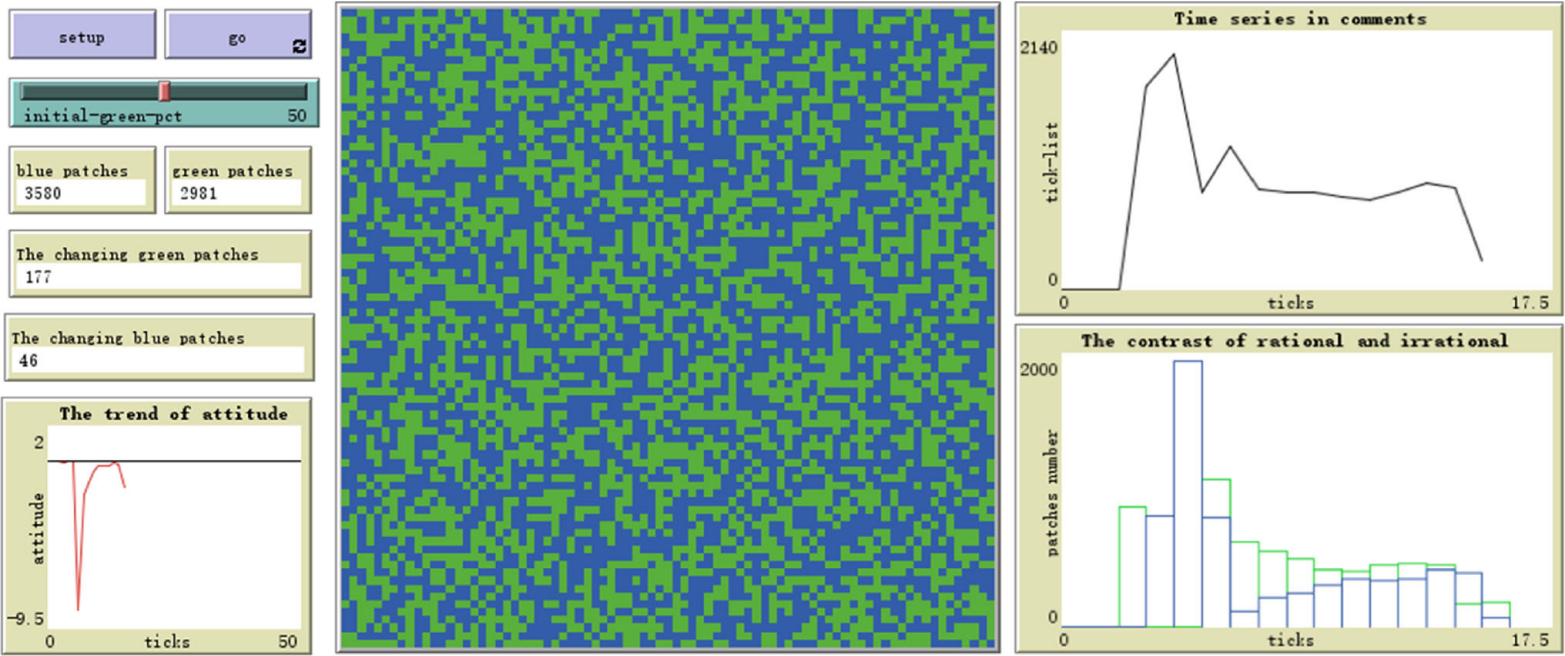
Basic interface of Ising model simulation. This is the NetLogo interface, which contains the controlling panels (upper left), monitoring plots (lower left), simulation panel (middle), and outcome plots (right)

In the NetLogo, the whole world consists of multiple patches, and each patch refers to one netizen. As we have 13,036 online discussions and each has two directions, the feasible number of agents should be around $6518=13036/2$. We set the world as the square grid, and the total area is, therefore, *S* = 81 × 81 = 6561 (Patches^2^). For each netizen, the cell space records the possible status of netizens. It can be one-dimensional, two-dimensional, or multi-dimensional [41]. We expand basic Ising model, which is one-dimensional, to simulate dynamic trends of online public opinions. At each time $t$, the netizen has two statuses, such as supportive (in blue) and oppositive (in green). Determined by behavioral rules, patches will change their status or not. At each time, we monitor quantitative global changes in blue and green patches. Meantime, we construct graphs and plots to observe dynamic trends of dynamic states for patches (Fig. 3).

### Neighborhood

Individuals do not live isolated, but within many social groups. Hence, they are always influenced by viewpoints of neighbors. To a considerable degree, the choices we make are conditioned on how others see and perceive this. In other words, our views of the world are heavily influenced by others, and we often seek out opinions of other whenever we need to make decisions [64]. In the Ising model, which is cellular automation model, the next states of cell greatly depend on current states of neighboring cells [65]. Because cells interact with neighbors, and we should model neighborhood structures. For Ising models, both Von-Neumann (*n* = 4 neighbors) and Moore (*n* = 8 neighbors) neighborhoods have been commonly used [65]. To capture more complex situations, we use the Moore neighborhood, which means that each agent on the patch has 8 neighbors whose viewpoints will influence current agents.

### The cellular states

The Ising model can be discrete in space, time, and state. Thus, each cell can be under one certain state at each time [66]. When the “NeiYuanWaiFang” case was released online, different netizens have different opinions, which produces polarization of individual viewpoints. For some agents of supporters, they believed that the society should not openly criticize the viewpoints of Ms. Fang Fang who just recorded some aspects of the Wuhan epidemic case. Besides, they claimed that the society should safeguard the independent spirit, critical consciousness, and even freedom of speech of Ms. Fang Fang; however, for others, this female writer did not know or reflect the real or whole situations of this epidemic, and event fabricated the facts out of thin air. She did not solve the main contradiction under the epidemic, but deliberately led to more secondary contradictions, which caused biased reports of Western medias. However, for people in discussions (whether for or against), there will always be some irrational discussions from Internet users. They used bullying, attack, and ridicule ways to express their viewpoints and suppress others at the same time. Thus, we supposed that cells have two different states, blue and green. Blue represents supportive discussions or comments, and we set value = 0. Green status refers to the oppositive discussions, and we set value = 1. The proportion of supportive and oppositive agents equals, and both account for about 50%. When the color changes from blue to green, the agent (cell) makes oppositive online postings. When the color changes from green to blue, the agent (cell) makes supportive postings or comments.

### Behavioral rules

The behavior rules of agents with neighborhoods guide individual decisions and behaviors, which are dynamical from one time ($t$) to the next ($t+1$) [67]. In this model, agents will change their viewpoints according to specific behavioral rules. Under the big data era, due to characteristics of anonymity and non-contactness, people are more willing to express their viewpoints and tell truths with more freedom [68]. As a macroscopic phenomenon, the number of online participants fluctuates with time, and the evolutionary dynamics of online public opinion cases contain four stages, such as preparation, outbreak, peak, and vanish [69]. According to the life cycle patterns of online cases, we have formulated relevant rules for online opinion conversions, to model the behavior rules or actions strategies for all agents. We use $S$ to refer to the strategy of agents. Then, ${S}_{t}$ refers to current action, and ${S}_{t+1}$ is the next action. In Eq. (1), the strategy update, ${S}_{t}\to {S}_{t+1}$, for all agents is largely shaped by the joint force of four behavior rules, such as $f1$, $f2$, $f3$, and $f4$.

1 $$ {S}_{t}\to {S}_{t+1}=\left(f1,f2,f3,f4\right).$$

Every event (online cases) needs to be prepared or organized, but this process is latent and invisible [70], which means that it takes a while for public opinions to explode. Then, online collective actions will gather enough momentum to attract public attention and discussion. The agents, used to model people, are smart and intelligent enough to adjust their strategies according to micro-level and macro-level situations. For most situations, individuals tend to use heuristics-rules of thumb [39]. When interacting with neighbors who are known to revenge untrustworthy ones, it is better for agents to keeping their commitments [71]. Thus, in the beginning or phase ${T}_{1}$, we set netizens are not easy to change their viewpoints. For the first rule in Eq. (2), the *N* is the total number of disagree neighbors of the agent. Only when more than half neighbors disagree with current viewpoints, can this agent change his or her opinions, under certain probability ${P}_{1}$.

2 $$f1:{\mathrm{Behavior}}_{\mathrm{it}}=\left\{\begin{array}{l} {\mathrm{unchanged}}, \, {\mathrm{if}} \,  N\le 4\\ P\left(\mathrm{chooses \, mainstream \, view}\right)={P}_{1}, \, {\mathrm{if}} \,  N>4\end{array}.\right.$$

Human beings are adaptive creatures, and they can learn norms, heuristics, and full analytical strategies from other agents, from feedbacks of the outside world [72]. The dominant viewpoint usually changes across time, and people will change their dynamic viewpoints to respond to them. When a new dominant viewpoint emerges, people may choose to align with these new viewpoints, whatever their original viewpoints are. Hence, at the ${T}_{2}$ phase, we set that if some neighbors choose to follow the mainstream (dominant) viewpoints, this agent $i$ will follow it under a certain probability ${P}_{2}$. If no one follows, neither does this agent follow them. This second rule $f2$ can be shown in Eq. (3), and N refers to the number of neighbors.

3 $$f2:{\mathrm{Behavior}}_{\mathrm{it}}=\left\{\begin{array}{l} {\mathrm{unchanged}}, \, {\mathrm{if}} \,  N<1\\ P\left(\mathrm{chooses \, mainstream \, view}\right)={P}_{2}, \, {\mathrm{if}} \,  N\ge 1\end{array}.\right.$$

As online collective event continues to ferment, individuals take more factors into account, including the number of participants involved, the benefits they have, heterogeneity of participants, and information communications [73]. Under the combined effect of these factors, the behavior rules become more complex, which then determine their own viewpoints. During this phase ${T}_{3}$, we discuss situations where individual viewpoints change under different situations. They become more prudent to change their viewpoints. If more than half neighbors follow the new mainstream viewpoint, the agent follow this new one. Otherwise, they will not change, even though there are already half neighbors who chose the new mainstream viewpoints.

4 $$f3:{\mathrm{Behavior}}_{\mathrm{it}}=\left\{\begin{array}{l}{\mathrm{unchanged}}, \, {\mathrm{if}} \,  N\ge 4\\ P\left(\mathrm{choose \, mainstream \, view}\right)={P}_{3}, \, {\mathrm{if}} \,  N<4\end{array}.\right.$$

In the final phase ${T}_{4}$, the heat of online discussion diminishes and stabilizes gradually. The strategy updating rule will be stricter, and it has more difficulties in strategy updates. As autonomous agents, it is much harder for them to change their viewpoints in the last phase ${T}_{4}$. Hence, agents will follow the new mainstreams, only when they see more than six neighbors having changed their viewpoints. If there are six or below neighbors changed, they will not change their viewpoints either.

5 $$f4:{\mathrm{Behavior}}_{\mathrm{it}}=\left\{\begin{array}{l}{\mathrm{unchanged}}, \, {\mathrm{if}} \,  N\le 6\\ P\left(\mathrm{choose \, mainstream \, view}\right)={P}_{4}, \, {\mathrm{if}} \,  N>6\end{array}\right..$$

To find optimal parameters, whose simulations can well match the data of real target cases, we set value ranges of the parameters and present optimal values of our agent-based model in Table 2. After multiple experiments and simulations, we finally determine the best parameters of behavior rules and start running the model under the best parameters. In Table 2, four stages (${T}_{1}$, ${T}_{2}$, ${T}_{3}$, and ${T}_{4}$) are divided qualitatively, based on observations of real cases. For this target case, ${T}_{1}$, ${T}_{2}$, ${T}_{3}$, and ${T}_{4}$ jointly form the whole life cycle trace of online public opinions, on a daily basis. Four action rules (${f}_{1}$, ${f}_{2}$, ${f}_{3}$, and ${f}_{4}$) play their roles on four specific stages (${T}_{1}$, ${T}_{2}$, ${T}_{3}$, and ${T}_{4}$). Within different four stages, we apply four value ranges in the second column of Table 2. Under multiple and repeated simulations, we obtain the optimal solutions, which can achieve highest matching degrees for both training data (the first half) and testing data (the other half). Four action rules is obtained from four value ranges in Table 2, such as (2–6), (1–5), (2–6), and (4–8). They are all covered by the whole range of (1–8) neighbors. Based on theoretical analysis and empirical data in the previous section, we have narrowed the whole range of [1, 8] to be smaller within four different stages. Four value ranges correspond to four probability ranges, which calculate the percentage within eight neighbors. During the whole process, each agent will shift (change) attitudes between positive and negative attitudes, according to how many agents (the number, percentage, or probability) opposing this current agent.

**Table 2:** The main parameters for this model

Stages	Value ranges	Probability ranges	Action rules obtained
${T}_{1}$ = (0–1 ticks)	${N}_{T1}$ = (2–6)	P1 = (1/4–3/4)	${f}_{1}({N}_{T1}^{*}=4 )$
${T}_{2}$ = (2–3 ticks)	${N}_{T2}$ = (1–5)	P2 = (1/8–5/8)	${f}_{2}({N}_{T2}^{*}=1 )$
${T}_{3}$ = (4–23 ticks)	${N}_{T3}$ = (2–6)	P3 = (1/4–3/4)	${f}_{3}({N}_{T3}^{*}=4 )$
${T}_{4}$ = (23 plus ticks)	${N}_{T4}$ = (4–8)	P4 = (1/2–2/2)	${f}_{4}({N}_{T4}^{*}=6 )$

### Features of our expanded model

Ising model is initially proposed to explain the phase transitions of magnetic materials, then it is largely expanded and becomes a useful method to explore criticality phenomena of multiple systems, such as the continuous quantum phase transitions [74], currency stability [75], and criticality of dynamics [76]. In this work, we expand Ising models in terms of two aspects: (a) we expand the application fields. Ising model has been applied to online collective actions, such as dynamical online opinions, which is an important topic in current computational social sciences. In traditional Ising models, each patch has two statuses (blue and green), and the status shifts between them at different time. Here, we extend the Ising model to pandemic-related online collective actions; (b) we can real-time monitor individual behaviors. However, this can only observe and describe the static and aggregated outcome. Here, we monitor each agent and record dynamical actions at each time. Therefore, we can know exact real-time actions or decisions of each agent. According, we can calculate frequencies of strategy changings and know the whole information. The distribution of actions (decisions) can be obtained; and (c) we combine big data mining and simulations to make optimizations. Via big data mining, we obtain the whole information of macro-level dynamics and patterns, as well as micro-level behaviors of individuals (netizens). Based on this, we know the whole big data process and features of the target online case, which is necessary to calculate the fitness of our model’s simulations. Finally, we are able to find at least one optimal solution, and the highest fitness can be, therefore, achieved.

## Outcomes and simulation fitness

China now has more than 900 million (mobile) internet users (netizens), and massive diverse opinions emerged have been witnessed on social media every day. Online social networking platforms [77], such as Weibo, Wechat, Douban.com, and TikTok, have greatly promoted the public discourses and inspired online engagement. The Douban.com platform is one of the most popular social medias and Chinese online communities. It has 4.83 billion public comments, attitudes and recognitions on films, music and books monthly. There are plenty of big data scorings and comments on cultural products and academic works, and it is one of the most authoritative and reference platforms in China. During the outbreak of COVID-19, most social media users also concentrate on this pandemic. Anything about this pandemic will cause massive attention and brings two polarizable comments, especially when public opinions are polarized online [78]. The ‘NeiYuanWaiFang’ case has been scored at 4.8 points (out of 10 points), because there are too many bipolar comments and discussions, which accounts for 86.8% of total. The score shows both polarized online opinions and stratification structures in current society of China. The agent-based modeling and simulations help to unveil the dynamics of online public opinions, and the optimal solution, which achieves the highest fitness, matters a lot. Thus, we use the algorithm, in Eq. (6), to solve or find optimal parameter setting. The term ${\mathrm{Par}}^{*}\left(\cdot \right)$ is defined as the optimal solution of our agent-based model and simulations. Under multiple simulations (*N* = 10,000), we calculate the differences $\Delta$ between real case and simulations, based on which we can finally obtain the optimal solution with the minimal difference $\Delta$. Practically, there are three steps. First, according to empirical observations of real cases and simulation outcomes, we divide the whole life process into four stages, such as T1 (ticks = 1), T2 (ticks < 4), T3 (ticks < 23), and T4 (ticks > 23). Second, we traverse related parameters in 10,000 simulations to find possible optimal solutions, ${\mathrm{Par}}^{*}\left(\cdot \right)$, which is preliminary and we can merely guarantee its validity. To further ensure the robustness, under the same parameter of ${\mathrm{Par}}^{*}\left(\cdot \right)$, we run simulations repeatedly for 10,000 times to acquire stable (average) outcomes. In this outcome section, we provide how and why our optimal solution of simulations can best match real outcomes of the target case. Under multiple scales, we all check the fitness of simulated outcomes to the observed big data (target case), such as micro, middle, and macro levels. At the macro level, we visualize the big data trends of supportive, oppositive and aggregated (total) comments. The time-serial characteristics include total and peak opinions and durations, which should be matched by simulations. At the middle-level, we visualize the whole-process distributions of subset or categorical comments, which should be matched by simulations. At the micro-level, we analyzed real big data of individual behaviors (decisions), compared to model’s simulations.

6 $${\mathrm{Par}}^{*}\left(\cdot \right)=\mathrm{ArgMin}\left(\Delta \right)=\mathrm{ArgMin}\left[{f}_{\mathrm{sim}}(\cdot )-{f}_{\mathrm{real}}(\cdot )\right].$$

### Micro-level matching

**Fig. 4:**
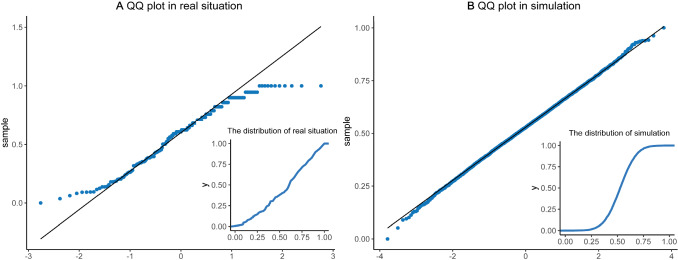
The normal distribution matching of individual behaviors. **A** provides the normal distribution checking (Q–Q plot) of individual behaviors for the real target case, and its subfigure visualizes empirical density function. It seems that most cases are normally distributed, except for extreme cases. **B** provides the normal distribution checking (Q–Q plot) of individual behaviors for the real target case, and its subfigure visualizes the empirical density function as well

The micro-level matching refers to the matching of individual behaviors, between simulations and real target case, which is a hard task because there are too many deviations directions. We apply the agents to simulate netizens online, and their behaviors should take on similar or same patterns, if our model holds. Therefore, we compare big data characteristics of individual behaviors under both real target case and simulations. In the statistical process control (SPC), many analysis methods are preconditioned on characteristics of normal distributions [79], such as Bayesian regression, logistic regression, KNN, and K-mean clusters. The skewness or non-normal data points can often be transformed into approximately normal distributions, using related logarithmic such as exponential transformations [80, 81]. For each netizen, we obtain a list of real-time big data in the number of personal comments. We collected more than 12,000 comments. About 667 people participated in the discussion, which can be obtained and identified by the ID of Douban.com. For the number of individual comments, the mean is 11.4225, and the SD is 35.4018, but original big data of real target case is not normally distributed. However, we can transform it and make it follow the normal distribution. It demonstrates in Fig. 4 that: (a) real case data. The data of original real case present the distribution shape with longer right tails. We follow three steps to obtain the normal distribution. First, we extract the square root of the real case data. Second, we calculate the reciprocal of the real case data. Last, we normalized the data again to facilitate the dimension removals. Therefore, the number of individual comments (behaviors) in real case can follow the normal distribution. In Fig. 4A, the normal Q–Q plot is applied to check the distribution, and the normality can be supported. Except for extremely small or large values, most data points are located at the 45° line where $y=x$. We provide accumulative empirical density curves (functions) in two Fig. 4A and B. For real target case, we have once observations data, but we have paralleled outcomes for multiple simulations; and (b) simulated data. We analyzed the big data of 10,000 repeated simulations, to explore their distributive features. The original data of simulations present a classic normal distribution. In Fig. 4B, we use the normal Q–Q plot to check the normal distributions. It well follows the normal distribution, and almost all data points are standing at the straight black line ($y=x$). The subfigure of Fig. 4B provides empirical accumulative density curve, which is infinitely close to the standard normal density curve. Comparing two distributions of real target case and simulated online collective behaviors, their distributive patterns are not only similar but also solvable to each other. As we have 10,000 times of observed data, the simulation data are more ideal and closer to the normal distributions. Although there are some differences between simulated agents and real individuals, the same distribution feature greatly supports the coincidence, consistence, and connections between real target cases and our agent-based model.

### Middle-level matching

**Fig. 5:**
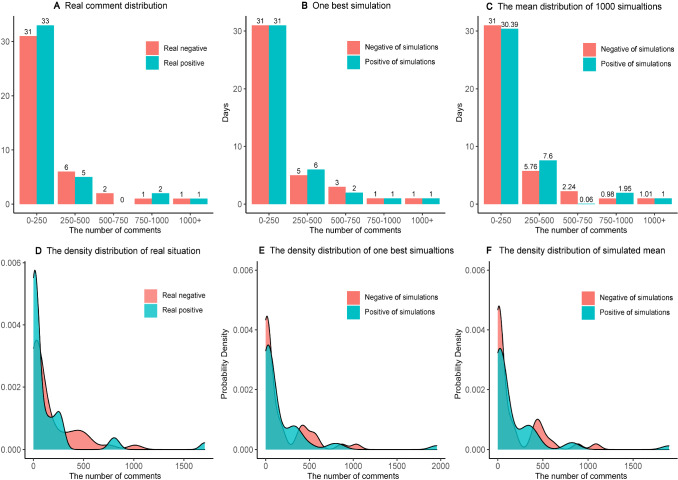
Distributions of supportive and oppositive comments. Subfigures **A**, **B**, and **C** visualize and compare discrete distributions of supportive and oppositive comments for both real case, one best simulation, and multiple simulations. Subfigures **D**, **E**, and **F** visualize and compare the continuous distribution forms (kernel density function), which have meanings across the whole range of *X* (time)

The opinions, sentiments, and related concepts (appraisal, attitude, evaluation, emotion, affect, and mood) are key indicators of human psychology and critical factors of group behaviors. For companies, organizations, and governments, it is necessary to analyze sentiment patterns from public opinions [64]. We not only need to browse a large number of websites to identify the information, but also identify the praise and derogation contained by information. So that we can real-time obtain online public opinions, deeply excavate online information, and analyze social networks (relationships), to find possible mechanism and dynamic trends [82]. If our model has successfully fitted sentimental and emotional distributions of real target case, the agent-based model will be well-supported, with the better accuracy and predictability. For this real case, we separate the entire text into single words. Based on the sentiment knowledge enhanced pre-training (SKEP) algorithm [83], we call the nature language analysis API from the Baidu AI platform. Then, we assess the sentiment scores and attitude polarity. We scrutinize potential semantic deviations and acquire the daily sentiment distributions to calculate the fitness of our model. To match the real case and simulations, we divide comments into five equal intervals, and each includes both 250 supportive and 250 oppositive comments. Figure 5A demonstrates the daily distributions of real comments, Fig. 5B shows the distribution of one (best) simulation, and Fig. 5C reflects outcomes of 10,000 simulations. It suggests that: (a) discrete distributions are well matched. Within the [0, 250] interval, we have real 33 supports and 30 oppositions in Fig. 5A. For one best simulation in Fig. 5B, we have 31 supportive and 31 oppositive comments. Assessing the 10,000 simulations, we have 30.39 supports and 31 oppositive, with tiny error bars. Hence, real target case and simulations can be well matched. Similarly, it seems that multiple simulations have better fitness than the single (best) simulation. Comparing the Fig. 5A and C, real target case and simulations (*N* = 10,000) are well matched at [250, 500] and even well matched at [500, 750], [750, 1000], and [1000, $\infty$]. For instances: under the [500, 750], real case has 0 supportive and 2 oppositive comments, and the multiple simulations (*N* = 10,000) has $0.04\approx 0$ supportive and $2.24\approx 2$ oppositive ones; under [750, 1000], the real case has 2 (supportive) and 1 (oppositive) comment, the multiple simulations have $1.95\approx 2$ supportive and $0.98\approx 1$ oppositive one; under [1000, $\infty$], the real has 1 and 1 comments, and simulations have 1 and $1.01\approx 1$ comment; and (b) continuous distributions are well matched. To avoid the grouping effects, kernel density functions (KDF) or curves are applied, to explore the continuous matching degree between real case and simulations. It suggests that the overall trends of both supportive and oppositive comments are well matched. For the oppositive comments, it has three peaks within the whole range of life cycle. For real case in Fig. 5D, the first peak is reached at the beginning (larger than 0), the second peak can be seen around *x* = 270, and the third happens when *x* = 750. For one best simulation in Fig. 5E, the first peak happens when *x* is slightly larger than 0, the second is seen around *x* = 280, and the third is around *x* = 750. This is also similar for multiple simulations. In Fig. 5F, the second peak happens when *x* = 280, and the third is seen when *x* = 750. Generally speaking, all kernel density curves have right tails, which can be seen in both simulations and real case.

### Macro-level matching of life cycles

**Fig. 6:**
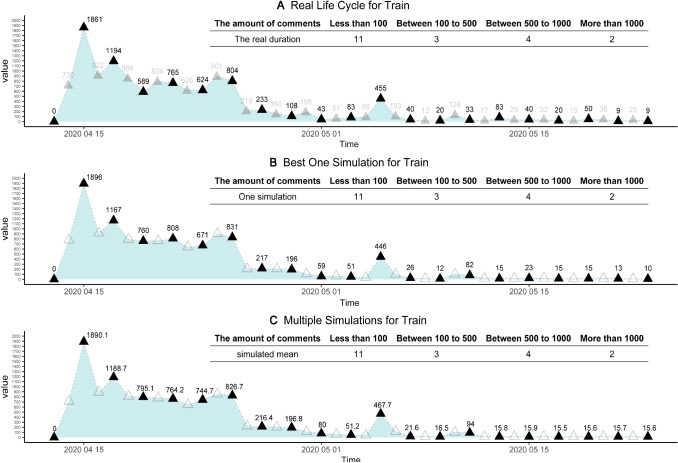
The fitness of the train data. Subfigure **A** indicates the real big data of the training sets, which is half of the time-serial data. Subfigures **B** and **C** elucidate the fitting outcomes of the best one simulation and multiple simulations, for the train dataset. Solid triangles represent the daily results of the real target case, which can be well fitted by training sets of simulations. Hollow triangles are unused values (test)

**Fig. 7:**
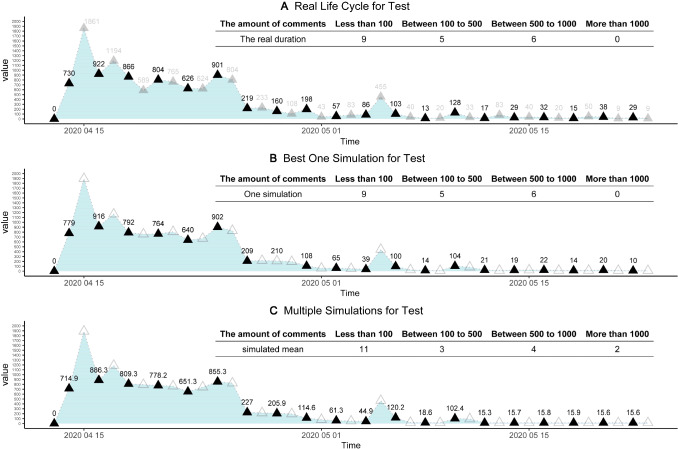
The fitness of the test. Subfigure **A** depicts the other half of the real dataset, which is the test dataset. Subfigures **B** and **C** elucidate the matching fitness of both best one simulation and multiple simulations. Solid triangles represent the fitted test data points, and hollow triangles are unused values (train)

**Fig. 8:**
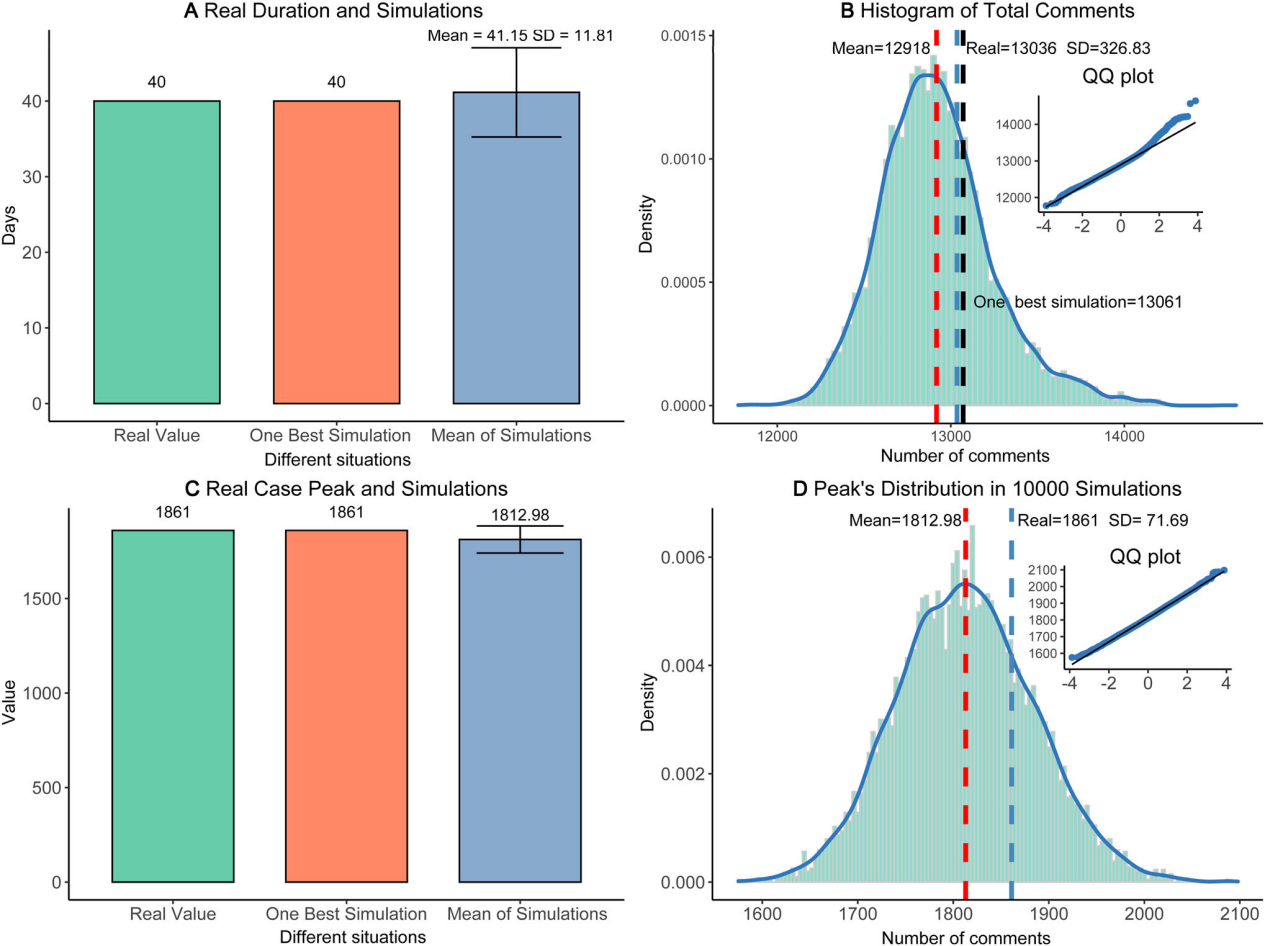
The life cycle durations matching. In subfigure **A**, we plot three durations in days of the real case, one best simulation, and multiple simulations; in subfigure **B**, we plot the distribution of the total number of total comments under 10,000 simulations, and compare the mean value (12,918) with the real case (13,036); in subfigure **C**, we compare three peak numbers of the real case, one best simulation, and multiple simulations; we further check the distribution of 10,000 peaks

**Fig. 9:**
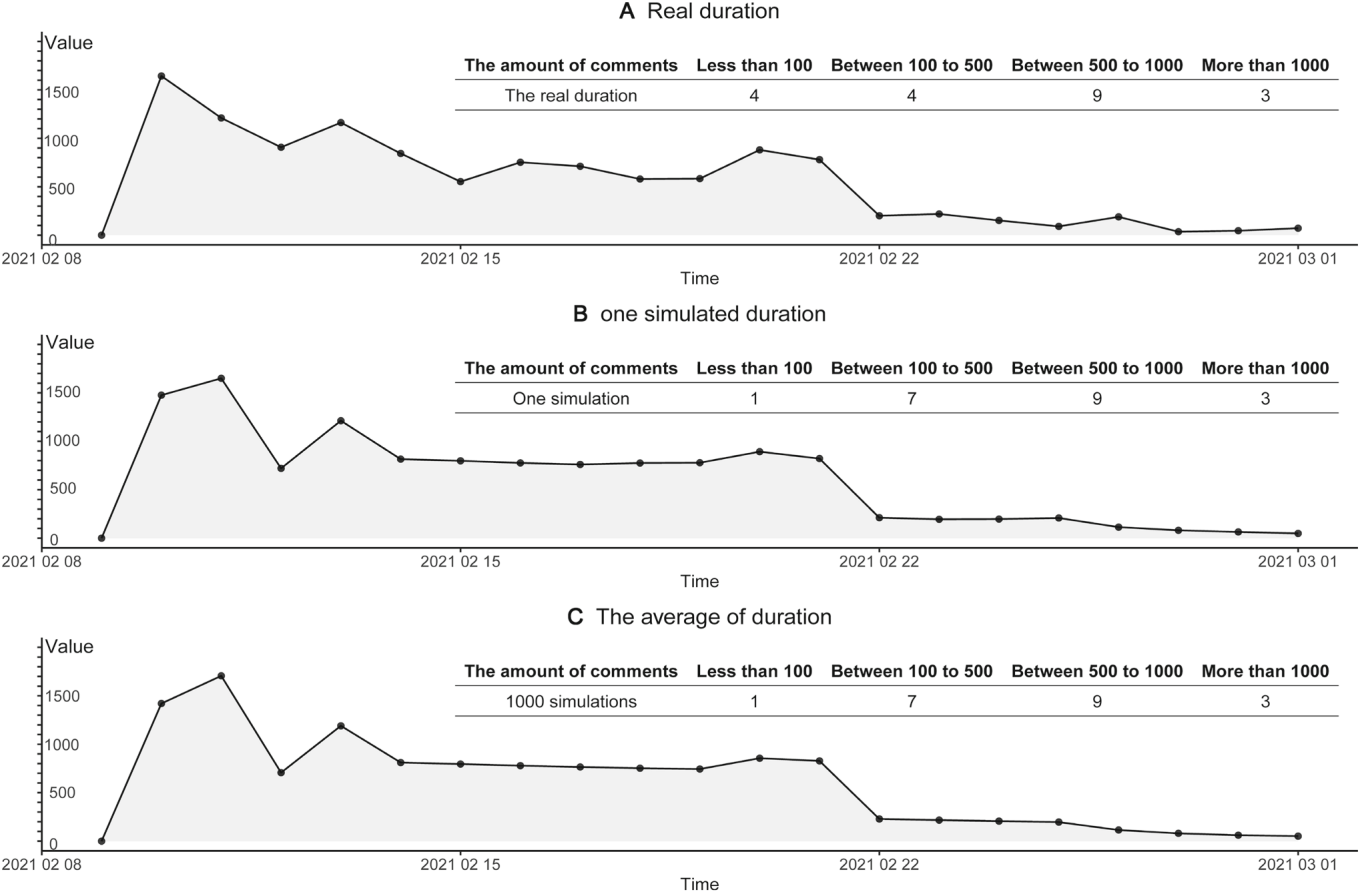
The outcomes of another NEW case. We plot the daily comment volumes to reflect life cycle trends of the real case in **A**, one best simulation in **B**, and multiple simulations in **C**. As well, we statistically evaluate the durations of the real case, one best simulation, and multiple simulations

The macro-level matching refers to life cycle similarities and fitness, which is universal for all online collective actions, including online public opinion dynamics.

(A) Life cycle stages matching. Unveiling the life cycles of online collective actions, the peak model shows the periodicity of online public opinions, which has four stages, such as prepare, outbreak, peak, and vanish [70]. The digital dairy called Fang Fang Diary was released online on April 12nd, which is the outbreak day. Then, online public opinions reached the peak of 1896 comments on April 15th, the beginning of peak stage. There were more than 500 comments daily before April 25th, the end of peak stage. For the next 5 days, the volume of daily comments was below 200. After May 15th, although there were some dispute-inducing comments, which ignited small battles with 400+ comments, it begins to vanish and online attention has inevitably shifted to others. Thus, the duration (span) is about 30+ days. There are no substantial trends after this life cycle period, although still some comments can be seen. According to life cycle theory, we divide it into four stages, the prepare (before the April 12), outbreak (April 12–April 15), peak (April 15), and vanish (after May 15). In our agent-based model and simulations, each tick refers to 1 day. According to real case observations and simulation ticks (steps), we define the stages of prepare (Ticks = 1), outbreak (1 < Ticks < 4), peak (Ticks < 23), and vanish (Ticks > 23). This is the necessary process of parameter calibration [84], which improves the fitness and validity of our model. The life cycle of our model can well fit the real target case, under multiple criteria, such as overall trends, total comments, comment peaks, and life cycle durations, besides of four stages.
(B) Life cycle curves (trends) matching of training and test datasets. We visualize three life cycle trends for the training set, to show perfect matching between real case and simulations. We divided the real big data that has been collected into two parts, such as the train set and test set. Each dataset comprised 50% of the original data, within the whole life cycle process, respectively. We adopted the training set to tune the model and examined the robustness of the model, and apply the hold-out cross-validation to avoid the probability of over-fitting. Figure 6 depicts the fitness of the best one simulation and outcomes of multiple simulations, for the training set. Obviously, using the first half of real data, our model shows a highly matching degree between reality and simulations, and the robustness of our model can be supported as well. Figure 6A shows the half data of the life cycle trends, as the training set of our model. Figure 6B shows the life cycle trend of best one simulation, and Fig. 6C shows the averaged outcomes of 5000 simulations. Comparing three trends in Fig. 6, it suggests that real case and simulations are well matched. First, they all reach the peak points at the same day, which is April 15th; Second, three peak stages end at the same day, which is April 25th; Third, in the late periods, three local peaks are reached at the same day, which is May 5th; Last, the life cycle trend is weak (death) after this local peak day. Besides of macro-level trends, daily details of comment distributions can be well matched as well. For real target case, we have 2 days with more than 1000 comments, 4 days with 500–1000 comments, 3 days with 50–100 comments, and 11 days less than 100 comments; the best one simulation and multiple simulations (*N* = 5000) provide same results as our real target case: 2 days with 1000+ comments, 4 days with 500–1000 comments, 3 days with 50–100 comments, and 11 days with 100− comments.
  Similarly, we use the second half data of the life cycle process (real target) as the test dataset, to further examine the fitness of our model. Figure 7 depicts the fitness of the best one simulation, and the outcomes of multiple simulations, for the test dataset. Figure 7B shows the outcome of best one simulation, and Fig. 7C shows the averaged outcome of 5000 simulations, for the test dataset. The test dataset is used to reduce possible over-fitting of the train dataset. Here, it seems that the matching between simulations and real target case is also satisfactory, for the test dataset, and our model has little over-fitting or over-training. For the test dataset of real target case, we have 6 days with 500–1000 comments, 5 days with 50–100 comments, and 9 days with 100− comments. Likewise, the best one simulation and multiple simulations (test dataset) have the same outcomes: 6 days with 500–1000 comments, 5 days with 50–100 comments, and 9 days with 100− comments. Therefore, for both train and test datasets, the Outbreak, Peak, and Vanish stages (the life cycle trends) can be well matched. Best one simulation (strict matching) and multiple simulations (perfect matching) support both validity and robustness of our model and optimal solution ${\mathrm{Par}}^{*}\left(\cdot \right)$.
(C) The life cycle durations matching of test sets. The life cycle periodicity, within the evolutionary process of online collective actions, has been revealed by the peak model as well as the life cycle theory of human behaviors [70]. As is indicated in Fig. 8, the durations are well matched between real target case and related simulations. From the prepare stage on April 13th to vanish stage on May 23rd, this online opinion case last for 40 days, until the day when we collect the data. After May 23rd, online comments become much weaker because most netizens pay attention to other cases. Therefore, this day is defined as the death of online cases. From April 13th to May 23rd, the life cycle span (duration) equals 40 days. Given optimal solution ${\mathrm{Par}}^{*}\left(\cdot \right)$, our simulations should match this real target span of 40 days. Figure 8A compares simulated durations with 40 days. For best one simulation, it is 40 days as well. For multiple simulations under optimal solutions (*n* = 10,000), the mean values 41.15, which is extremely close to 40 days. Considering the SD as 11.81, there is no difference between the real case and simulations. Hence, the spans of simulations can be deemed the same as real target case.
(D) The matching of online comments. Based on our agent-based model and simulations, simulated comments can also match real target case. It includes: (a) total comments matching. First, we pre-process the raw data, excluding invalid text such as ‘…’, ‘?’, or meaningless symbols. We in total obtain 13,035 comments for the real target case, within the whole life cycle. For our agent-based model, the daily number of comments is defined as how many times of color change (strategy update) for agents at all ticks. Hence, we obtain the number of comments for each simulation. For the best simulation, the total is 13061 comments in Fig. 8B, which is infinitely close to 13,036 in reality. For 10,000 simulations in Fig. 8B, we plot the distribution of total comments, as well as the kernel density estimation (KDE) curve [85]. The mean is 12918.44 and the standard deviation (Nesdale, #313) is 326.83, which well matches the total amount of 13,036 comments for the real target case; and (b) comment peak matching. For the real target case, the peak number of comments is witnessed on April 15th. On this peak day, many users of Douban.com expressed their opinions and argued with others, and we have 1861 comments. For the best simulation, the peak number in Fig. 8C is exactly 1861, which is a perfect match. In Fig. 8D, we compare the peaks of real target cases with multiple simulations, and there seem to be no differences between them. For multiple simulations (*N* = 10,000), the mean of peaks is 1812.98, the standard error (SE) is 0.73, which can be shown as the error bar in Fig. 8B. Besides, the standard deviation (Nesdale, #313) is 71.69 in Fig. 8D, which implies that the simulated mean value (1812.98) has no significant differences from the real peak (1861). To further compare them, the kernel density estimation (KDE) was used to construct the distribution of 10,000 simulated peaks. In Fig. 8D, the smooth density curve is also visualized, and the Q–Q normal plot is applied to verify normal distributions. Obviously, this is normal distribution, because most data points stand at the straight line. Therefore, our model and simulations are well consistent with reality.
(E) The generality test. We add another example, namely “Yang Li Case”, to examine the generality of our model. There is a famous talk show actress, Ms. Yang Li, who advocates feminism and attacks (or makes fun of) men in China. However, she once endorsed the clothing brand HLA, which mainly sells men’s clothing. Then, a lot of online criticize and discussions happens on Zhihu.com, the largest Q&A community in China (similar to Quora). We use Python to obtain the dynamic number of online discussions. This case in 2021 lasted from February 10th to March 1st, the duration is 20 days. By the last day, the total number of discussions about Yang Li is 11613, and the peak number is 1643. This case is applied to do the robustness tests. Figure 2 shows that the matching between reality (Fig. 2A) and simulations (Fig. 2B, C) is ideal. For the best one simulation in Fig. 2B, it has a total number of 12,092, a peak of 1650, and the duration of 20 days. We select the best parameters based on the possible parameters range in Table 2 to match the data of real cases and acquire the final the stages T1 (0–1 ticks), T2 (2–3 ticks), T3 (4–13 ticks), and T4 (after 23 ticks). For 1000 simulations, it has a total number of 12,050, a peak number is 1706. Thus, our model also fits this “Yang Li Case”, which supports the robustness of fitness (Fig. 9).

## Conclusions and discussion

Under social media and the big data era, we often witness online collective actions, which are caused by possible and multiple topics. The internal dynamics include idea interactions and (supportive-oppositive) opinion struggles. We model the dynamics of opinion-forming, using agent-based model and simulations. Eventually, we obtain the optimal solution of simulations with the best fitness achieved. The existence of the optimal solutions supports the strongest explaining power, based on which it is feasible to explain, describe, and even predict life cycles of online opinions. We have several conclusions and discussions: (a) our model can best fit the life cycles of real target case. Ising model is initially proposed to explore natural science phenomena. We apply this to model online public opinions (collective actions), which are typical topics in social sciences. According to real situations, we expand the Ising model in terms of several aspects. More attention is paid to individual behaviors, and this micro-level process can be recorded and analyzed. Based on the agent-based modeling of this expanded Ising model, the results of simulations can match the big data trend of the real case. The life cycle trends of simulations and the real target case can be well matched, which reinforces both validity and robustness of our agent-based modeling. The criteria include amount, peak, duration, etc.; (b) our expanded model provides new methods to predict the real target cases. Giving the mechanism of individual behaviors can be seized or the optimal solution can be found or solved, our model can be used to describe, explain, and forecast the life cycle process of online public opinions. For specific real target cases, we are able to find the correspondingly optimal solutions and check their robustness by repeated simulations. Ising models also have been used to predict the voting processes and outcomes [86]. Our model upgrades traditional research methods of online public opinions [83, 87, 88] and it provides new perspectives for emergency management online. Using this model, we can predict the whole process of online public opinions, which helps online emergency responses; and (c) limitations. Of course, there are some limitations to this work. We only selected a recorded incident that occurred during the epidemic, and the number of comments is limited. In future, we will select more real cases of online public opinions for simulation to further improve both effectiveness and robustness of the model.
